# Does Water, Waste, and Energy Consumption Influence Firm Performance? Panel Data Evidence from S&P 500 Information Technology Sector

**DOI:** 10.3390/ijerph17145206

**Published:** 2020-07-19

**Authors:** Liliana Nicoleta Simionescu, Ștefan Cristian Gherghina, Ziad Sheikha, Hiba Tawil

**Affiliations:** Department of Finance, Bucharest University of Economic Studies, 010374 Bucharest, Romania; liliana.simionescu@fin.ase.ro (L.N.S.); ziad.sheikha@gmail.com (Z.S.); hibatawil95@gmail.com (H.T.)

**Keywords:** water, waste, energy, panel data

## Abstract

This paper aimed to investigate the impact of water, waste, and energy consumption on firm performance for a sample of enterprises that belong to the S&P 500 Information Technology sector over the period of 2009–2020. The quantitative framework covered both accounting (e.g., return on assets—ROA; return on common equity—ROE; return on capital—ROC; return on invested capital—ROIC) and market-based measures of performance (e.g., price-to-book value—PB), alongside firm and corporate governance specific variables. By estimating multivariate panel data regression models, the empirical results provided support for a negative impact of total water use on PB but a positive effect on ROA. With reference to the total waste, the econometric outcomes revealed a negative influence on the entire selected performance measures, whereas total energy consumption did not reveal any statistically significant influence.

## 1. Introduction

In the context of the augmented degradation of the environment and competitive market circumstances, managers are concerned as to how green practices can be employed to enhance organizational performance [1]. Jiang et al. [2] emphasized that green entrepreneurial orientation has positive effects on environmental and financial performance. Thus, in order to meet sustainable development goals, enterprises purse investments for novel and inclusive low-carbon products, dropping the carbon footmark of their manufacturing operations, setting emanation decrease targets, and enhancing their energy productivity [3]. Hence, the promotion of corporate social responsibility (henceforth “CSR”) has put pressure on companies regarding their relationships with the environment, society, and economy [4]. However, CSR is not an emerging phenomenon [5]; rather, it pinpoints that businesses cannot be detached from ethics, and every stakeholder should be taken into consideration in all activities of the company [6]. Even if CSR policy is an extra and voluntary cost that affects core business operations, it is a benefit to shareholders, as socially responsible companies become more attractive to stakeholders and society [7]. Moskowitz [8] suggested that socially responsible companies perform better than conventional corporations. Additionally, Gallego-Alvarez et al. [9] found that European companies that have adopted CSR practices are better-founded than other companies. Therefore, Mishra and Suar [10] confirmed that the CSR practices of stakeholders would be beneficial to firms. Companies should comprehend that CSR activities are not a burden but a way of investment because the expenses paid in the short term provide higher profits and a better reputation in the long term [11]. Rjiba et al. [12] claimed that CSR investments counterbalance the adverse effect of economic policy uncertainty on enterprise performance. Bhattacharyya and Rahman [13] proved a positive connection amid mandatory CSR spending and Indian firm performance. Nehrt [14] found that earlier investors in pollution dropping tools registered higher profit growth than subsequent investors. Thus, socially responsible companies could reduce their transaction costs [15] and may become more effective over time [16]. Additionally, higher profits in the short term and sustainable development in the long term [17] will lead to improved firm performance. At the same time, CSR exerts a robust impact on insolvency risk mitigation [18].

Prior research has focused on energy efficiency and financial performance in Korea [19], China [20], Spain and Slovenia [21]; energy efficiency and productivity/exporting in Latin America [3], Ethiopia [22], and India [23,24]; waste and enterprise performance in Japan [25] and the US [26,27]; emissions and firm performance in Europe [28,29,30], Japan [25], and the US [26,31,32]; and other diverse countries worldwide [33]. Additionally, earlier studies have focused on a wide range of industries [2,27,30,32,33,34,35,36]; manufacturing firms [21,24,25,31,37,38,39]; manufacturing and industry [40]; manufacturing, service, and IT organizations [1]; mining and manufacturing companies [41]; energy companies [19,20,42,43]; fossil fuel-related and non-fossil-fuel-related industries [44]; and industrial and commercial firms [45]. Nevertheless, industries intensely diverge in their emanations, whilst a company’s environmental performance substantially hinges on its industrial positions [46]. Hence, a single industry analysis would more precisely catch the related environmental proactivism [27]. This paper attempts to investigate the impact of water, waste, and energy consumption on firm performance out of the US Information Technology sector. Accordingly, the novelty of current paper emerges from approaching the technological sector, which reveals several distinct particularities. Product lifespan is commonly reduced, as new products with boosted performance continuously appear with superior complexity [47]. Hence, the generation of electronic waste has increased to 44.7 million metric tons yearly, being comparable to nearly 4500 Eiffel towers, whereas merely 20% of it is attested to be gathered and recycled [48]. The production of semiconductor, which are crucial elements in technology products, involves huge amounts of ultra-pure water to circumvent the dirt of electronic tools. For instance, a common semiconductor manufacturing plant employs two-to-four million gallons of ultra-pure water daily [49]. Nevertheless, semiconductor wastewater usually comprises several intractable chemicals like organic solvents, acids, bases, salts, heavy metals, and fine suspended oxide particles, among other organic and inorganic compounds [50]. In addition, the energy used to produce digital devices is substantially higher than the energy used during their functioning [44]. However, tech giants are seeking to lessen carbon and waste footprints and to encourage reprocessing, alongside water conservation [51].

The remainder of the paper is organized as follows. The following section discusses prior literature, and the third section reveals the data and research methods. The fourth section shows the quantitative outcomes. The last section emphasizes the main findings and their implications, study limitations, and future research avenues.

## 2. Related Literature, Theoretical Framework and Hypotheses Development

### 2.1. Prior Research on Corporate Environmental Responsibility–Corporate Financial Performance Association

European Commission [52] defines CSR as “actions by companies over and above their legal obligations to society and the environment.” However, CSR has no certain definition according to Dahlsrud [53], who found that the prevailing meanings are categorized into the following dimensions: (1) environmental dimension—the relationship with natural environment; (2) social dimension—the relationship with the society; (3) economic dimension—financial side and business operations; (4) stakeholder dimension—all parties that have a relation with the company; and (5) voluntariness dimension—practices and activities not issued by laws. Crifo et al. [54] explored over 10,000 French companies and defined three dimensions for CSR as (1) environmental, (2) human resources, and (3) relations with customers and suppliers.

Each organization has primary and support activities to perform the business, which have both positive and negative effects on society and environment. Unlike sustainability, CSR operates on a short-term vision [55]. It shows that a company has realized externalities that may affect society and should be accounted for in their daily decisions [56]. Thereby, the organization will be able to find solutions that benefit society without harming the environment, while also generating profits [57]. Because of the awareness of consumers of corporate activities, some companies have designed CSR programs to compensate for the bad effects caused by their operations [58]. In this vein, corporate environmental policies have been planned to mainly fulfill regulatory necessities and to pacify societies [59]. Goll and Rasheed [60] found that the environment has a moderating effect on the relationship between social responsibility and firm performance. Famiyeh [61] emphasized the importance of CSR investments in order to improve operational competitive capabilities in regard to cost, quality, flexibility, delivery and global performances.

The form of the association amid corporate social performance and financial performance (questions regarding linear or non-linear relationships and the type of non-linearity) is not definite [62]. By surveying 32 studies, Molina-Azorin et al. [63] concluded mixed outcomes, but found the prevalence of a positive influence of the environment on financial performance. Hence, there have been contradictory findings regarding the effect of CSR on firm performance. Stakeholder theory advises that social performance positively influences financial performance because it augments the contentment of diverse stakeholders and thus the external reputation, resulting in better financial performance [44]. Therefore, socially responsible companies tend to promote longstanding associations with stakeholders instead of maximizing immediate profit [64]. Saeidi et al. [65] found that reputation and competitive advantage mediate this relation. A competitive advantage could be gained through different channels such as good communications with all stakeholders, creating new business opportunities, and developing working settings [66]. Sardana et al. [67] demonstrated that environmental sustainability practices influence firm performance through institutional viewpoints (regulatory, normative, and cognitive).

In contrast, the trade-off theory postulates that CSR exerts an opposing effect on firm performance [68]. Friedman [69] argued that the only purpose of a business is to maximize shareholders’ wealth, but the importance of other stakeholders was not claimed. Hemingway and Maclagan [70] found that the adoption of CSR policy is a way to hide fraudulent and unethical activities. As such, Kim and Im [71] hypothesized that a corporation that is more involved in CSR is also interested in circumventing taxes via long-term tax planning. Ucar and Staer [72] argued that enterprises situated in zones with high corruption display decreased levels of CSR scores. Additionally, Crisóstomo et al. [73] found a lack of a statistically significant relationship amongst CSR and financial accounting performance for Brazilian companies. Nevertheless, Hoepner and Yu [74] emphasized that CSR may have different effects on firm performance depending on the industry. Bernal-Conesa et al. [75] showed a significant impact of CSR on technological companies’ performance. Additionally, Bernal-Conesa et al. [76] showed a positive association between CSR integration and enterprise reputation, alongside a positive relationship between internal improvement and technological firms’ performance.

Table 1 provides a summary review of the prior findings of the impact of corporate environmental responsibility on corporate financial performance.

### 2.2. Earlier Studies on the Impact of Technological Innovation on Company Performance

The mass-production of the 20th century was carbon-intensive and extraction-based, raising central queries about the sense of the development [80]. Hence, aiming to dissociate economic activity from the consumption of limited resources, the concept of circular economy focused on recycling, effectiveness, and productivity, emerged along with the goal of preserving goods in operation for longer [81]. Additionally, ecological modernization theory suggests that sustained industrial progress, instead of unavoidably continuing to damage the atmosphere, provides the supreme choice of running away from the worldwide ecological challenge [82]. Andries and Stephan [83] argued that ecological innovations lead to better financial performance because they permit companies to lessen rubbish removal and basic material cost, raise product value and company rivalry, and decrease public and community burden, along with providing support to figure upcoming rules which increase opponents’ relative prices.

Table 2 reveals an overview of preceding research on the influence of technological innovation on company performance.

### 2.3. Previous Literature Regarding the Influence of Water, Waste and Energy Consumption on Firm Performance

Good management theory argues that good management practices are connected to corporate social performance because relations with all stakeholders are enhanced, resulting in improved global performance [90]. The closely associated natural resource-based view assumes that a company can accomplish sustainable competitive rewards via the assignment of its resources and capabilities in eco-friendly business actions [91]. Accordingly, a U-shaped curve is claimed by the natural resource-based view, whereas the slack resource view contends that the connection is reliant on the level of financial resources of the company [39]. Wen and Lee [37] found that Chinese manufacturing enterprises augmented their financial performance and productivity after gathering environmental labeling certification due to the intervention effect. Özbuğday, Fındık, Özcan, and Başçı [40] documented a positive effect of resource efficiency investments on small and medium-sized enterprises’ sales growth. Agovino et al. [92] found that recycling rates of packaging, e-waste, and bio-waste positively influenced enterprise competitiveness in Europe. Additionally, Cucchiella, Gastaldi and Miliacca [29] found that the implementation of an environmental management system (henceforth “EMS”), along with control of emanations, may lead to profits via a surge in demand and efficiency. Nishitani and Kokubu [38] confirmed that environmental performance boosts added value by directly amending the production process and indirectly amending rising demand through unveiled ecological evidence. Additionally, institutional theory specifies that enterprises under strong institutional burden will acquire legitimacy by demonstrating good environmental performance [43]. Mungai, Ndiritu, and Rajwani [45] concluded that the implementation of voluntary environmental management systems is related to the betterment of ecological performance in Kenya. Hence, an increasing number of corporations worldwide have become conscious of the value of protecting the natural environment and have agreed to adopt an EMS for improved conformity, avoidance of environmental occurrences, and to reveal the figure of an ecologically reactive entity [93]. Eng et al. [94] revealed the cost saving provided by implementing a wastewater treatment and recycle within the semiconductor industry.

Porter [95] claimed that improved ecological performance may be valuable for companies as long as pollution is an indication of economic inefficiency. Furthermore, Porter and Linde [96] underlined the compromise between ecology and economy, wherein the trade-offs are the societal gains that ensue from stringent environmental rules against the business’s particular aims for avoidance and cleansing which determine upper prices and low competitive capacities. However, Hart [97] contended that in the developed states, many corporations are “going green” because they recognize that pollution may be lessened, whereas revenues can be enlarged at once. Therefore, an extensive amount of studies has been performed on the impact of ecological strategies on firm performance due to the increased significance of this dispute in environmental management. Based on a strategic management viewpoint, Lundgren and Zhou [98] argued that companies that strengthen energy productivity are expected to raise output, diminish environmental pressure, encourage ecological investment, reduce carbon tax burden, and augment market standing. Moreover, energy efficiency enhancement may be a significant policy for augmenting competitiveness since it lessens functioning charges [23]. However, if ecological performance is driven by guidelines, a productivity loss is registered. Lee et al. [99] revealed that environmental performance exhibits a positive influence on return on common equity (ROE) and return on assets (ROA) for Korean enterprises. For the case of Korea, Moon and Min [19] explored 19 enterprises belonging to non-metal industries and 17 companies from the food sector and found a significant link between energy efficiency and financial performance. Fan, Pan, Liu, and Zhou [20] investigated six Chinese high-energy-consuming industries and showed a positive influence of energy efficiency on firm performance, as measured by return on equity, return on assets, return on investment, return on invested capital, and return on sales, but they also found a lack of association with Tobin’s Q. Makridou, Doumpos, and Galariotis [28] found that a decline of CO2 emissions and number of allowances exerts a positive effect on firms’ profitability. On the contrary, Abdisa [22] underlined a negative effect of power disruptions on firm productivity, namely an increase of costs.

Pons, Bikfalvi, Llach, and Palcic [21] explored a sample of Spanish and Slovenian manufacturing enterprises and argued that the use of energy and material saving technologies does not show a significant impact on return on sales. Iwata and Okada [25] concluded that waste emissions do not influence firm performance for Japanese manufacturing firms. Fakoya [41] explored 64 companies quoted on the Johannesburg Stock Exchange Socially Responsible Investment Index and found a lack of statistically significant association among investment in hazardous solid waste lessening and return on assets. Gonenc and Scholtens [44] employed a global sample of enterprise in both fossil fuel-related and non-fossil-fuel-related industries, and they found a lack of association between the environmental performance of fossil fuel firms and return on equity.

Table 3 provides a brief review of the outcomes of earlier studies regarding the influence of water, waste, and energy consumption on firm performance.

Henceforth, based on the particularities of the Information Technology sector, the following hypotheses are postulated:

**Hypothesis** **1** **(H1).** *As long as the manufacturing process in the Information Technology sector requires large quantities of ultrapure water and the associated wastewater contains unwilling chemicals, the effect on firm performance is negative.*

**Hypothesis** **2** **(H2).** *As long as the amount of electronic waste is augmented but the recycle rate is lower, the impact of waste on firm performance is negative.*

**Hypothesis** **3** **(H3).** *Since the production of tech devices is energy-intensive and, hence, a noteworthy contributor to the world’s greenhouse gas emissions, the effect of energy consumption on firm performance is negative.*

## 3. Research Methodology

### 3.1. Sample Selection and Data Collection

The sample comprised 71 technological companies covered by the S&P 500 index over the period of 2009–2020, and the data were gathered from Bloomberg. The selected variables are described in Table 4. Consistent with earlier research, in order to measure firm performance, the quantitative investigation comprised both accounting measures—such as return on assets [2,19,20,25,26,30,32,33,34,35,39,41,77,78,85,86,87,88,99,100,101], return on common equity [19,20,25,30,32,33,39,42,44,86,99], return on capital [19,20], and return on invested capital [25]—and market-based measures of performance like price-to-book value [34]. Additionally, several measures of firm characteristics were included in order to counteract any bias and error that may have distorted the association among selected variables [41].

Corporate liquidity measures are covered driven by that fact that enterprises that suffer from a low liquidity level may attempt to lifting their disclosure level of the CSR and voluntary actions [102]. In line with earlier research [13,24,26,29,31,33,34,36,38,41,42,43,77,84,86,88,90,99,100,101], indebtedness was included here because of the fact that a minor financial risk motivates an enterprise to implement technical innovation because it is convenient to persuade lenders and attract capital [18]. Fakoya [41] noticed that indebtedness measures the amount that a firm is funded by debt capital and shows the level to which external resources finance ecological investments. Sun and Cui [18] asserted that CSR raises company cash flow, lessens income instability, generates firm value, and engenders insurance-like assets that shelter companies from default. Cash-flow variables were defined as they have been in earlier studies [13,41,86] since the likelihood of earnings management is greater in corporations with a high excess free cash flow [64].

Furthermore, as in studies by Bhattacharyya and Rahman [13] and Fakoya [41], profitability was included. However, depending on the approached theory (e.g., stakeholder theory or trade-off), the relationship among profitability and CSR is inconclusive. With reference to taxation, Kim and Im [71] found that corporations concerned with CSR hinder tax circumvention, but passive CSR-implicated firms do not attempt tax avoidance. In regard to dividend policy, a higher payout decreases the accessible cash for executives and deters them from over-investing in CSR, but it also signals the firm’s reputation [103].

Like prior studies [3,13,20,24,25,26,27,30,31,33,35,39,42,44,46,77,78,79,83,85,90,91,99,100,101], firm size was included since large companies generally register a superior profit level compared with small corporations [20]. Larger firms are supposed to invest more in ecologically responsive machineries, as they are expected to have more funds and because they accept higher litigation risks [79]. Waddock and Graves [90] claimed that smaller enterprises may not show many evident socially responsible actions compared to larger companies because, as they develop, they entice more outside consideration and have to overtly comply to stakeholder requests. Hence, the resource-based view argues that larger corporations gain more from ecological innovations, particularly in compliance to guidelines or sector ethics codes, whereas stakeholder theory contends that smaller firms benefit due to the effect of customer demand [83].

Corporate governance variables were included following [34,45,46,79,104] because resource-based theory postulates that enterprises must have superior management abilities in order to follow proactive environmental policies [79]. For instance, Liu [104] documented that companies with superior female board representation register less ecological judicial proceedings. However, since the implementation of pollution-lessening approaches brings many challenges, executives are inclined to circumvent such strategies and assign funds to more traditional investments. Thus, an incentive mechanism should be employed [46]. Accordingly, board and executive compensation were covered similarly by Li, Ngniatedema, and Chen [34], as well as by Berrone and Gomez-Mejia [46].

### 3.2. Quantitative Framework

In line with prior studies [20,21,24,25,41,44,88,99], our quantitative approach was grounded in panel data regression models. In order to examine the impact of water, waste, and energy consumption, alongside firm and corporate governance-specific variables, on firm performance, we estimate the following pooled ordinary least squares regression models:

ROA_it_ = α_0_ + β_1_TWU_it_ + β2TW_it_ + β3TEC_it_ + β_4_CorporateLiquidity_it_ + β_5_CorporateIndebtedness_it_ + β_6_Cash-flow_it_ + β_7_Profitability_it_ + β_8_CorporateTaxation_it_ + β_9_DividendPolicy_it_ + β_10_FirmSize_it_ + β_11_CorporateGovernance_it_ + u_it_ (1)

ROE_it_ = α_0_ + β_1_TWU_it_ + β2TW_it_ + β3TEC_it_ + β_4_CorporateLiquidity_it_ + β_5_CorporateIndebtedness_it_ + β_6_Cash-flow_it_ + β_7_Profitability_it_ + β_8_CorporateTaxation_it_ + β_9_DividendPolicy_it_ + β_10_FirmSize_it_ + β_11_CorporateGovernance_it_ + u_it_ (2)

ROC_it_ = α_0_ + β_1_TWU_it_ + β2TW_it_ + β3TEC_it_ + β_4_CorporateLiquidity_it_ + β_5_CorporateIndebtedness_it_ + β_6_Cash-flow_it_ + β_7_Profitability_it_ + β_8_CorporateTaxation_it_ + β_9_DividendPolicy_it_ + β_10_FirmSize_it_ + β_11_CorporateGovernance_it_ + u_it_ (3)

ROIC_it_ = α_0_ + β_1_TWU_it_ + β2TW_it_ + β3TEC_it_ + β_4_CorporateLiquidity_it_ + β_5_CorporateIndebtedness_it_ + β_6_Cash-flow_it_ + β_7_Profitability_it_ + β_8_CorporateTaxation_it_ + β_9_DividendPolicy_it_ + β_10_FirmSize_it_ + β_11_CorporateGovernance_it_ + u_it_ (4)

PB_it_ = α_0_ + β_1_TWU_it_ + β2TW_it_ + β3TEC_it_ + β_4_CorporateLiquidity_it_ + β_5_CorporateIndebtedness_it_ + β_6_Cash-flow_it_ + β_7_Profitability_it_ + β_8_CorporateTaxation_it_ + β_9_DividendPolicy_it_ + β_10_FirmSize_it_ + β_11_CorporateGovernance_it_ + u_it_ (5)

where α_0_ denotes the intercept; β_1_–β_11_ are the coefficients to be estimated; ε is the disturbance term; i = 1, 2, …, 71, and t = 2009, 2010, …, 2020; TWU is total water use; PB is price-to-book value; ROIC is return on invested capital; TEC is total energy consumption; and TW is total waste. Additionally, in order to alleviate heteroscedasticity, we considered a robust standard error in addition to the baseline form.

## 4. Empirical Findings and Discussion

### 4.1. Summary Statistics and Correlations

**Figure 1:**
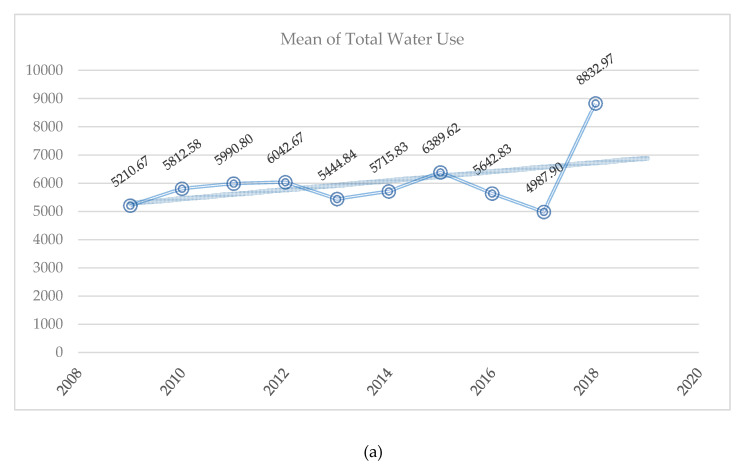

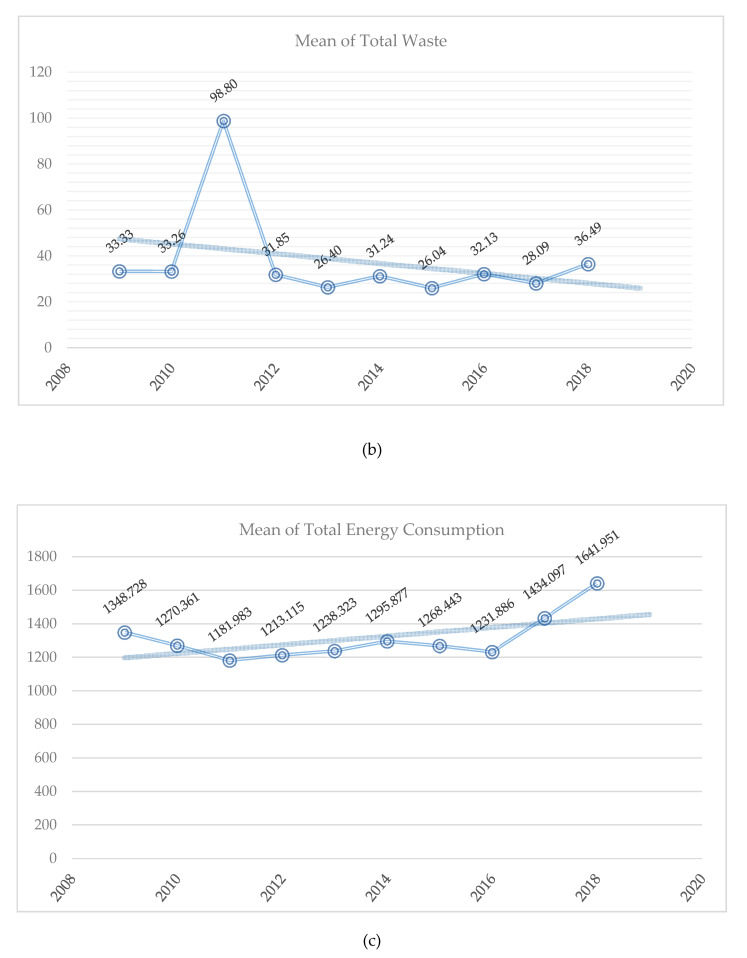
The annual means of (**a**) total water use, (**b**) total waste, and (**c**) total energy consumption. Source: Authors’ work.

Table 5 reveals the summary statistics for the variables used in the empirical research. We noticed that total water use registered the highest mean values, whereas total waste showed the lowest mean values. Figure 1 plots the annual means of total water use, total waste, and total energy consumption. As long as a small number of companies reported the data for the last two years, 2019 and 2020 are not covered in Figure 1. An increasing trendline of total water use and total energy consumption was noticed, and this was facilitated by the fact that in order to produce and power the related equipment, data hubs, or facilities requirements, a huge amount of electricity is needed. Therefore, the energy footprint of the Information Technology industry is projected to exhaust around 7% of global electricity [105]. Additionally, the International Energy Agency [106] advised that deprived of novel strategies, the energy spent by information and communications tools along with consumer electronics will double by 2022 and upsurge threefold by 2030 to 1700 terawatt hours, which will threaten the diligence of raising energy security and lessen the emanation of greenhouse gases. However, with reference to total waste, a decreasing trendline occurred because many large tech corporations are leaders in environmental responsibility [107].

Similar prior studies [21,24,26,28,31,33,34,35,41,42,99,101], correlations amongst variables are pointed out in Table 6. High correlations between explanatory variables are not reported, except for total debt to capital (TDC) and total debt to total assets (TDTA) (0.86), which showed that multicollinearity is less likely to be an issue. As such, we noticed weak correlations between water, waste, and energy consumption and firm performance—this was positive in case of TEC, negative with reference to TW, and mixed for TWU.

### 4.2. The Outcomes of Panel Data Regression Models

The regression results regarding the impact of water, waste, and energy consumption, alongside firm and corporate governance-specific variables, on accounting performance, in regard to return on assets and return on common equity are presented in Table 7. The coefficients of total water use revealed a positive and statistically significant impact only on ROA, which is in line with the view “do well by doing good” [90], as well as the win–win circumstance of Porter [95]. Consequently, the econometric outcome failed to support Hypothesis 1. As new factories are assembled, they are motivated to integrate internal water recycling methods in order to prevent major costs of ecological conformity and modernization in the future [47]. Several companies from the Information Technology sector employed water recycling facilities, which put them in a good marketing place and gave them a confident corporate image. These enterprises may benefit from premium pricing and augmented sales due to market acceptability and better social consent [63], thus leading to the registering of better performance due to the acquisition of more customers [102].

However, the coefficients related to total waste were found to negatively influence ROA, thus providing support for Hypothesis 2. Porter and Linde [96] found that damaging materials being released into the environment is an indication that resources have been exploited partly, inadequately, or unproductively. Hence, corporations should undergo extra actions that increase cost but generate no value for clients. Additionally, Lahouel, Bruna, and Zaied [77] argued that greater ecological regulations and severe national environmental guidelines adversely influence companies’ performance by involving supplementary, irredeemable charges.

With reference to total energy consumption, rather than expected from Hypothesis 3, the impact on firm performance, as measured by ROA and ROCE, was not statistically significant, as opposed to some prior studies [19,20] but in line with the study of Pons, Bikfalvi, Llach, and Palcic [21]. Nevertheless, consistent with the work of Li, Ngniatedema, and Chen [34], we found that the effect of energy on firm performance may not be instantaneous and might take more time for an enterprise to feel its influence. Nehrt [14] argued that enterprises, when deprived of the necessary time to assimilate new technologies, face time compression diseconomies that hinder them from enjoying all of their investments’ returns.

In line with earlier studies [33,41], the presence or absence of multicollinearity was investigated by means of the variance-inflation factors (henceforth “VIFs”). In this vein, Table 8 shows the mean VIFs. Since the related figures were much below the threshold value of 10, we noticed that the empirical outcomes were not affected by multicollinearity issues.

Table 9 reports the estimates regarding the impact of water, waste, and energy consumption on market-based performance. The empirical outcomes provided support for a negative influence of total water use and total waste on PB, which was consistent with the work of Cordeiro and Sarkis [27] and Hart and Ahuja [32]. Therefore, the results supported Hypotheses 1 and 2. In the short term, investors have found environmental measures as possible expenses or penalties, thus leading to adverse effects on firm performance [101]. Nevertheless, even if the corporations in the Information Technology sector attempt to be ecologically proactive, the opposite outcome does not indicate the loss of money in the long run. Generally, short-term imperfections related to pro-environment policies are more than compensated for by long-term benefits [27].

Likewise, analogous to the outcomes provided in Table 7, total energy consumption was not found to reveal any statistically significant effects on firm performance; hence, Hypothesis 3 could not be maintained. According to Hart and Ahuja [32], there is a delay between the launch of emanations lessening efforts and the occurrence of benefits. Initially, training and machineries should be funded, after which the renegotiation of supply clearance agreements and internal restructuring is needed.

Additionally, the mean VIFs reported in Table 10 show that there were no concerns for multicollinearity.

### 4.3. Robustness Checks

Aiming to assess the robustness of the empirical findings, we employed ROC and ROIC as alternative measures of firm performance. The estimation results reported in Table 11 reinforce the negative impact of total waste on firm performance, alongside the lack of statistically significant influence of total energy consumption. From an agency viewpoint, the adverse effect of waste may be explained by the opportunistic behavior of managers who may use resources to follow their own objectives instead of investing in environmental projects [63]. In regard to total water use, the related coefficients provided support for the absence of any association with firm performance. Consistent with the work of Wang [88], diverse ecological technologies were found to exert dissimilar effects on firm performance. Hypothesis 2 was confirmed, but neither Hypotheses 1 or 3 could be supported.

The mean VIFs with reference to estimation outcomes are reported in Table 12. Hence, as long as the related values of VIFs are below 10, there are no issues regarding multicollinearity.

## 5. Conclusions

This study investigated the influence of total water use, total waste, and total energy consumption on firm performance for a sample of enterprises in the S&P 500 Information Technology sector over the period of 2009–2020. The results showed mixed evidence in the case of total water use, namely a negative impact on price-to-book value, but a positive effect on return on assets. In regard to the total waste, the empirical findings provided support for an adverse influence on firm performance. Nevertheless, total energy consumption did not reveal any statistically significant impact on enterprise performance.

The research has implications for policymakers and company managers. With reference to water consumption, the wastewater from the manufacturing process should be suitably handled and discharged [94]. In this respect, transparency regarding water use, alongside industry regulations for reporting, should be imposed. As climate change is amplifying and water risks are becoming obvious, rigorous guidelines concerning water productivity and dismissal are essential.

With respect to waste, even if tech corporations are regularly concerned for environmental safety, electronic waste registers the highest growth. For instance, as the technology begins to move to 5G, there are many devices that are unsuited to novel technical ideas and will become outdated, leading to an increase in electronic waste. In this vein, policymakers should continuously track electronic waste statistics in order to lessen its emergence, avoid illegitimate removal and inappropriate handling, encourage recycling, and generate works in the refurbishment and recycling sectors [44]. Additionally, laws that forbid electronics from ordinary trash should be designed. Likewise, the recycling responsibly of company managers should be strengthened. Additionally, industrial development bonds should be considered in order to finance the establishment of electronic recycling facilities. Managers should be more concerned with waste prevention, along with end-of-pipe treatment [26]. Furthermore, directors should permanently exhibit a positive attitude toward sustainability through constant investments in green innovation [87]. Hence, ecological modernization should be considered by executives so as to support companies in accomplishing waste lessening or removal, resource recovering and dematerialization, and the reuse of goods [86].

Concerning energy, the enhancement of power management and productivity may generate supplementary returns, whereas saving energy can help reduce global warming [23]. Manufacturers should focus on producing equipment that needs very little power, apart from extending the battery life of portable tools. Thus, the related components should operate more efficiently to make sure that energy is only used when needed and to the desired scope. Apart from energy-intensive manufacturing procedures, the very short lifespan of many devices should not be disregarded. With the extended lifespan of digital tools, their related energy would not be an urgent matter. As such, the ecological footprint of digital technology may be lessened by tackling technical outmodedness [108]. In order to move their manufacturing processes forward, enterprises should shift from fossil fuels to renewable energy such as solar, wind, or hydropower.

The study had some limitations. First, the quantitative analysis covered large technological companies included in the S&P 500 index. Nevertheless, small and medium corporations should be considered because they also lead to ecological deprivation. As long as the positive consequences of environmental proactivism are not immediate [27], a lag regression model should be considered. In view of the rising concern about pollutant emission drops, future lines of research may extend the current investigation by exploring the impact of carbon releases on firm performance.

## Figures and Tables

**Table 1:** Prior studies regarding the corporate environmental responsibility–corporate financial performance relationship.

Author(s)	Time Span	Sample	Selected Variables Regarding Corporate Environmental Responsibility	Empirical Methods	Outcomes
Lahouel et al. [77]	2005–2017	61 French listed firms.	ASSET4 ESG environmental score.	Panel smooth transition regression.	Inverted-U association between environmental performance and Tobin’s Q. Inverted-V association between environmental performance and return on assets (ROA).
Tzouvanas, Kizys, Chatziantoniou and Sagitova [39]	2005–2016	288 European manufacturing corporations.	Greenhouse gas emissions.	Quantile regressions.	Positive effect of environmental performance on financial performance.
Robaina and Madaleno [78]	2008–2016	17 Portuguese sectors.	Carbon intensity by sector.	Panel feasible generalized least squares regression.	A higher amount of released greenhouse gases drives a higher level of financial performance until a particular level, from which the association reverses.
Horvathova [30]	2004–2008	Czech firms.	Environmental performance based on European Pollutant Release and Transfer Register.	Panel data fixed effects and random effects regression models.	Augmented firm’s emanations lessen firm profitability in the 2 year lag period but meliorates in the 1-year lag period.
Elsayed and Paton [35]	1994–2000	227 UK public limited companies.	Community and environmental responsibility scores developed by Management Today survey.	Panel data fixed effects and random effects regression models.	Environmental performance has little or no influence on financial performance.
Nishitani, Jannah, Kaneko and Hardinsyah [36]	1–28 February 2011	100 Indonesian Enterprises.	Greenhouse gas emissions reduction, pollution emissions reduction, and environmental management score.	Instrumental-variables ordered-probit model.	Enterprises can augment profit by merely dropping greenhouse gas emissions. Companies can boost profit by lessening greenhouse gas releases through lowering production costs, not via rising sales.
Alvarez [33]	2007, 2008, 2009, 2010	89 firms from Diverse nations worldwide.	Variation in CO2 releases.	Multiple regression analysis.	CO2 emanations changes negatively influenced ROA in 2007, whereas for the rest of the period, the impact was not statistically significant.
King and Lenox [31]	1987–1996	652 US manufacturing companies.	Total emissions, relative emissions, industry emissions, regulatory stringency, permits.	Panel data fixed effects and random effects regression models.	Pollution decrease is associated with Tobin’s Q.
Cordeiro and Sarkis [27]	1992	523 US companies.	Fugitive non-point air emissions, stack or point air emissions, discharges to receiving streams and water bodies, underground injections on-site, releases to land on-site, discharges to publicly owned treatment works, other off-site transfers, on-site and off-site energy recovery, on-site and off-site recycling, on-site or off-site treatment, and non-production releases.	Multiple regression analysis	Environmental proactivism negatively influenced industry analysis at 1-and 5-year earnings per share.
Hart and Ahuja [32]	1989–1992	127 companies out of S&P 500.	Emissions reduction.	Multiple regression analysis.	Emanation drops improve firm performance more for companies with upper emissions levels than for firms with reduced releases levels.
Clarkson et al. [79]	1990–2003	242 US firms.	The inverse of pollution propensity as toxic discharges in pounds scaled by the cost of goods sold.	Three-stage least squares analysis.	Positive relationship amongst environmental performance and financial performance.

Source: Authors’ work based on the literature review.

**Table 2:** Earlier research concerning the technological innovation-company performance linkage.

Author(s)	Time Span	Sample	Selected Variables Regarding Technological Innovation	Empirical Methods	Outcomes
Hojnik and Ruzzier [84]	2013	223 Slovenian enterprises.	Low energy consumption, recycle, reuse, and remanufacture material; usage of cleaner technology to generate savings and avoid pollution; decrease of the emanation of hazardous substances and waste; and reduction of the use of raw materials.	Structural equation model.	Eco-innovation positively influences profitability, productivity, and market share.
Xie et al. [85]	2013	209 Chinese listed firms	Green process innovation, green product innovation, green image, and green subsidies.	Multiple regression analysis.	Green process innovation and green product innovation meliorate financial performance.
Lin et al. [86]	2011–2017	163 worldwide automotive companies	Green innovation strategy.	Generalized method of moments.	Positive influence of activities related to green innovation strategy on corporate financial performance,
Rezende et al. [87]	2006–2015	356 multinational enterprises	The share of green patents relative to total patents.	Fixed-effect regressions.	Green innovation intensely, positively influences financial performance in the long run.
Wang [88]	2011–2013	248 US corporations	Share of investment in pollution control, energy efficiency, green design, low-carbon energy, and management systems.	Ordinary least squares regression.	Raising the portion of low-carbon energy and pollution control technologies negatively influences return on assets.
Fernando et al. [89]	2014	95 Malaysian companies	Product innovation, process innovation, service innovation, organizational/management/business model innovation, and any innovation applying a new technology.	Structural equation model.	Eco-innovation and service innovation capabilities influence sustainable business performance in corporations employing green technology.

Source: Authors’ work based on the literature review.

**Table 3:** Earlier literature on water, waste, and energy consumption-firm performance association.

Author(s)	Time Span	Sample	Selected Variables Regarding Environmental Performance	Empirical Methods	Outcomes
Hassan [42]	2013, 2014, 2015 and 2016	420 energy enterprises in the Organisation for Economic Co-operation and Development (OECD) states.	Renewable energies as measured by feed-in-tariff, grant, investment tax credit, and green certification.	Panel data fixed effects regression models.	Renewable energy incentive strategies positively influence financial performance.
Li, Ngniatedema and Chen [34]	2012–2013	434 US top listed firms.	Energy productivity, carbon productivity, water productivity, waste productivity, and green reputation.	Regression analysis.	Green initiatives positively influence financial performance, but the effect is varied and diverges for different sectors.
Subrahmanya [23]	2001–2002	40 Indian firms.	Energy cost.	Regression analysis.	Small firms where labor efficiency was greater and energy intensity was minor realized better returns relative to those where work productivity was lesser and energy intensity was higher.
King and Lenox [26]	1991–1996	614 US manufacturing companies.	Total emissions, waste generation, waste prevention, waste treatment, and waste transfer.	Panel data fixed effects regression models.	Firm emissions negatively influence financial performance.
Sahu and Narayanan [24]	2005–2013	34 Indian manufacturing firms.	Energy intensity.	Panel data fixed effects and random effects regression models.	Positive impact of energy intensity on profitability, except for natural gas grouping.
Lee and Gokalp [100]	2006–2011	363 firms out of Fortune 500.	Green energy use.	Two-stage Heckman model.	Positive relation between future Tobin’s Q and green energy use.

Source: Authors’ work based on the literature review.

**Table 4:** Variables’ descriptions.

Variables	Description	Unit of Measurement	Types
Variables regarding firm performance			
ROA	Return on Assets	% of Avg. Total Assets	Dependent
ROE	Return on Common Equity	% of Common Equity	Dependent
ROC	Return on Capital	% of Capital	Dependent
ROIC	Return on Invested Capital	% of Invested Capital	Dependent
PB	Price-to-Book Value	Times	Dependent
Variables concerning water, waste and energy consumption			
TWU	Total Water Use	Gallons Per Year	Independent
TW	Total Waste	Million Pounds	Independent
TEC	Total Energy Consumption	Billion U.S. Dollars	Independent
Variables concerning corporate liquidity			
CURR	Current Ratio	Times	Independent
QR	Quick Ratio	Times	Independent
Variables regarding corporate indebtedness			
TDTA	Total Debt to Total Assets	% of Total Assets	Independent
TDC	Total Debt to Capital	% of Capital	Independent
Variables regarding cash-flow			
CFFI	Cash Flow from Investing Activities	Billion U.S. Dollars	Independent
CFFF	Cash Flow from Financing Activities	Billion U.S. Dollars	Independent
Variables regarding profitability			
OM	Operating Margin	% of Revenues	Independent
Variables concerning corporate taxation			
ETR	Effective Tax Rate	% of Taxable Income	Independent
Variables related to dividend policy			
DY	Dividend Yield	% of Stock Price	Independent
DPR	Dividend Payout Ratio	% of EPS (Earnings Per Share)	Independent
Variables regarding firm size			
TA	Total Assets	Million U.S. Dollars	Independent
EMP	Number of Employees	Monetary Unit	Independent
Variables regarding corporate governance			
WOMFRC	Percentage Women in Workforce	% of Total Workforce	Independent
BRDCOMP	Total Board Compensation Paid	Million U.S. Dollars	Independent
EXECOMP	Total Executive Compensation Paid	Million U.S. Dollars	Independent

Source: Authors’ work.

**Table 5:** Descriptive statistics.

Variables	Obs.	Mean	Std. Dev.	Min	Max
ROA	747	8.999584	8.117018	−47.2279	35.91574
ROE	707	21.87037	26.12471	−111.184	194.3815
ROC	638	16.6797	17.73134	−69.4031	194.1054
ROIC	743	15.86454	21.05043	−68.4936	341.5322
PB	770	6.320321	8.451899	0.55133	137.2181
TWU	224	5959.881	12730.71	65.608	96,000
TW	222	72.17348	538.4997	0.126	7920.23
TEC	281	1307.811	1803.64	12.6966	8320
CURR	752	2.389425	1.678565	0.621066	11.84813
QR	752	1.847757	1.454174	0.031196	10.75744
TDTA	752	20.64898	16.76182	0	96.91215
TDC	748	34.519	41.34024	0	585.9127
CFFI	770	−1770.71	6049.508	−56,274	34,724
CFFF	770	−1663.94	6568.258	−102,977	14,324
OM	762	18.6102	15.39459	−105.203	66.151
ETR	642	29.26245	58.60471	0	1366.327
DY	426	2.297216	2.685658	0.038786	32.91229
DPR	745	44.57395	230.5365	0	5425.455
TA	756	23,447.74	44,475.18	51.369	375,319
EMP	695	40,888.55	76,820.8	375	492,000
WOMFRC	235	31.48438	8.272572	0.068919	59.6
BRDCOMP	681	2.721229	1.480708	0.06227	14.11653
EXECOMP	692	33.98355	39.63852	0.471579	436.6071

Source: Authors’ computations. Notes: For the definition of variables, please see Table 4.

**Table 6:** Correlation matrix.

**Variables**	**TA**	**EMP**	**CFFI**	**CFFF**	**OM**	**ETR**	**TDC**	**TDTA**	**CURR**	**QR**	**DY**	**DPR**
TA	1.00											
EMP	0.19	1.00										
CFFI	−0.66	−0.04	1.00									
CFFF	−0.66	−0.18	0.03	1.00								
OM	0.41	−0.17	−0.41	−0.28	1.00							
ETR	0.15	0.01	−0.09	−0.14	0.26	1.00						
TDC	0.33	0.22	−0.05	−0.26	−0.11	−0.06	1.00					
TDTA	0.29	−0.17	−0.08	−0.16	0.05	−0.09	0.86	1.00				
CURR	−0.26	−0.46	0.16	0.24	0.24	−0.09	−0.20	0.02	1.00			
QR	−0.17	−0.40	0.11	0.20	0.23	−0.12	−0.16	0.05	0.98	1.00		
DY	−0.12	−0.10	0.07	0.16	−0.22	−0.31	0.11	0.16	−0.01	0.01	1.00	
DPR	−0.06	−0.22	0.12	0.13	−0.05	−0.10	0.04	0.25	0.23	0.27	0.72	1.00
EXECOMP	0.69	0.18	−0.48	−0.45	0.39	0.12	0.03	−0.04	−0.23	−0.17	−0.11	−0.13
BRDCOMP	0.07	0.35	0.08	−0.03	−0.12	0.04	0.08	−0.07	−0.15	−0.13	0.15	−0.07
TEC	0.42	0.36	−0.32	−0.24	0.43	0.25	0.24	0.12	−0.27	−0.25	−0.02	−0.05
TWU	0.02	−0.04	−0.15	−0.04	0.44	0.21	−0.23	−0.14	−0.10	−0.17	0.05	−0.01
TW	−0.07	0.01	0.07	0.04	−0.17	−0.04	0.06	0.06	−0.14	−0.13	−0.11	−0.13
WOMFRC	−0.10	0.26	0.08	−0.03	−0.20	−0.02	0.01	−0.08	−0.22	−0.28	0.06	−0.01
PB	0.27	0.65	−0.02	−0.41	0.24	0.20	0.37	0.13	−0.16	−0.13	−0.24	−0.07
ROE	0.27	0.73	−0.13	−0.33	0.19	−0.04	0.44	0.11	−0.27	−0.24	−0.16	−0.26
ROA	0.19	0.16	−0.26	−0.24	0.72	0.02	−0.26	−0.22	0.05	0.02	−0.21	−0.18
ROC	0.15	0.63	−0.15	−0.23	0.29	−0.01	−0.11	−0.29	−0.21	−0.20	−0.21	−0.24
ROIC	0.04	0.62	−0.09	−0.15	0.24	0.00	−0.23	−0.38	−0.18	−0.18	−0.24	−0.21
**Variables**	**EXECOMP**	**BRDCOMP**	**TEC**	**TWU**	**TW**	**WOMFRC**	**PB**	**ROE**	**ROA**	**ROC**	**ROIC**	
EXECOMP	1.00											
BRDCOMP	0.16	1.00										
TEC	0.28	0.18	1.00									
TWU	0.06	0.00	0.69	1.00								
TW	−0.06	−0.04	−0.02	−0.03	1.00							
WOMFRC	−0.11	0.01	−0.06	−0.04	0.06	1.00						
PB	0.15	−0.03	0.24	−0.11	−0.12	0.21	1.00					
ROE	0.23	0.06	0.31	−0.04	−0.11	0.16	0.82	1.00				
ROA	0.30	−0.06	0.13	0.27	−0.18	0.05	0.45	0.50	1.00			
ROC	0.22	0.00	0.08	0.00	−0.14	0.25	0.71	0.77	0.79	1.00		
ROIC	0.12	−0.04	0.00	0.00	−0.14	0.29	0.67	0.66	0.73	0.96	1.00	

Source: Authors’ computations. Notes: For the definition of variables, please see Table 4.

**Table 7:** The outcomes of panel data pooled regression models regarding the influence of water, waste, and energy consumption, alongside firm and corporate governance-specific variables, on return on assets and return on common equity.

Variables	ROA					ROCE				
	Model 1	Model 2	Model 3	Model 4	Model 5	Model 1	Model 2	Model 3	Model 4	Model 5
WOMFRC	0.07	0.14	0.10	0.07	0.00	0.57	0.56	0.49	0.30	0.16
	(0.0523)	(0.0737)	(0.0851)	(0.0480)	(0.0572)	(0.2344) **	(0.2538) **	(0.3273)	(0.2564)	(0.2269)
	(0.0398)	(0.0595) **	(0.1004)	(0.0423)	(0.0606)	(0.1725) **	(0.1789) **	(0.2440) **	(0.2355)	(0.1593)
EXECOMP	−0.02	−0.01	0.04			−0.08	−0.04	0.05		
	(0.0090) **	(0.0136)	(0.0263)			(0.0403) **	0.04	(0.1000)		
	(0.0069) **	(0.0111)	(0.0150) **			(0.0201) **	(0.0307)	(0.0773)		
TDC	−0.06	−0.07		−0.03		0.20	0.35		0.48	
	(0.0123) **	(0.0168) **		(0.0114) **		(0.0998) **	(0.1128) **		(0.1356) **	
	(0.0159) **	(0.0141) **		(0.0172)		(0.1249)	(0.1216) **		(0.1928)	
CURR	−0.35	0.03	1.26	−0.71		−4.54	−1.57	−0.82	−2.86	
	(0.2865)	(0.4058)	(0.3839) **	(0.3285) **		(1.2507) **	(1.3531)	(1.5034)	(1.7257)	
	(0.3145)	(0.4343)	(0.5867) **	(0.2762) **		(1.3375) **	(1.1999)	1.16	(1.4550)	
DY	0.20	−0.13	0.21	0.38		0.08	−0.32	0.43	0.76	
	(0.1745)	(0.2247)	(0.2203)	(0.14531) **		(0.7643)	(0.7480)	(0.8344)	(0.7509)	
	(0.2780)	(0.4630)	(0.3831)	(0.2082)		(1.1200)	(1.5337)	(1.1014)	(1.0688)	
OM	0.33			0.51		0.58			0.86	
	(0.0395) **			(0.0583) **		(0.1738) **			(0.3220) **	
	(0.0473) **			(0.0481) **		(0.1772) **			(0.2029) **	
ETR	−0.00	−0.01	−0.01	−0.00	−0.11	−0.04	−0.05	−0.05	−0.05	−0.43
	(0.0034) **	(0.0045) **	(0.0046) **	(0.0028) **	(0.0424) **	(0.0155) **	(0.0158) **	(0.0176) **	(0.0154) **	(0.1672) **
	(0.0012) **	(0.0022) **	(0.0020) **	(0.0010) **	(0.0436) **	(0.0084) **	(0.0099) **	(0.0084) **	(0.0101) **	(0.1233) **
CFFI		−0.00					−0.00			
		(0.0000)					(0.0002)			
		(0.0000)					(0.0001)			
TA		0.00	−0.00	−0.00	0.00		−0.00	−0.00	−0.00	0.00
		(0.0000)	(0.0000)	(0.0000)	(0.0000) **		(0.0000)	(0.0000) **	(0.0000)	(0.0000) **
		(0.0000)	(0.0000)	(0.0000)	(0.0000) **		(0.0000)	(0.0000)	(0.0000)	(0.0000) **
EMP		−0.00					0.00			
		(0.0000)					(0.0000) **			
		(0.0000)					(0.0000) **			
TWU			0.00		0.00			−0.00		0.00
			(0.0000)		(0.0000) **			(0.0001)		(0.0001)
			(0.0000)		(0.0000) **			(0.0000)		(0.0000)
CFFF			−0.00					−0.00		
			(0.0000)					(0.0002)		
			(0.0000) **					(0.0002)		
TEC				−0.00					0.00	
				(0.0002)					(0.0011)	
				(0.0002)					(0.0010)	
TW				−0.00	−0.00				−0.00	−0.00
				(0.0004)	(0.0005)				(0.0024)	(0.0021)
				(0.0001)	(0.0002) **				(0.0006) **	(0.0005) **
BRDCOMP					−0.12					0.63
					(0.2322)					(0.9071)
					(0.2298)					(1.0326)
TDTA					−0.07					0.32
					(0.0362) **					(0.1542) **
					(0.0366) **					(0.1981)
QR					0.57					−1.35
					(0.3617)					(1.4418)
					(0.2755) **					(1.4116)
DPR					−0.01					−0.16
					(0.0170)					(0.0662) **
					(0.0156)					(0.0509) **
_cons	7.36	13.06	2.14	1.46	11.56	13.26	9.76	14.05	−12.16	23.77
	(2.2914) **	(3.2513) **	(3.4227)	(2.3557)	(2.9314) **	(10.2432)	(11.1556)	(13.6247)	12.30	(11.5303) **
	(1.8387) **	(2.5978) **	(4.1340)	(1.9984)	(3.1628) **	(8.4906)	(7.3012)	(9.4575)	(10.6812)	(9.8845) **
R−sq	0.5545	0.2839	0.3006	0.6579	0.4351	0.3409	0.3608	0.2031	0.3918	0.2539
Obs.	120	115	84	88	107	113	108	78	82	102

Source: Authors’ computations. Notes: Superscripts ** indicate a statistical significance level of 5%. The first figure between brackets shows the standard deviation, while the second figure between brackets shows the robust standard deviation. For the definition of variables, please see Table 4.

**Table 8:** Variance inflation factors (VIFs) for the panel data pooled regression models in regard to the influence of water, waste, and energy consumption, alongside firm and corporate governance-specific variables, on return on assets and return on common equity.

Variables	ROA					ROCE				
	Model 1	Model 2	Model 3	Model 4	Model 5	Model 1	Model 2	Model 3	Model 4	Model 5
WOMFRC	1.14	1.2	1.17	1.19	1.51	1.15	1.21	1.18	1.26	1.54
TWU			1.03		1.08			1.03		1.08
TEC				1.55					1.6	
TW				1.04	1.03				1.04	1.03
EXECOMP	1.07	1.39	2.04			1.09	1.38	2.02		
BRDCOMP				1.17					1.17	
CFFF			1.9					1.91		
CFFI		1.65					1.72			
TDC	1.09	1.15		1.18		1.16	1.32		1.49	
TDTA					1.19					1.16
CURR	1.23	1.43	1.22	1.54		1.2	1.4	1.27	1.58	
QR					1.39					1.42
DY	1.12	1.06	1.06	1.09		1.12	1.06	1.06	1.11	
DPR					1.16					1.16
EMP		1.34					1.32			
TA		2.27	2.81	1.59	1.18		2.49	2.84	1.72	1.22
OM	1.22			1.89		1.2			2.11	
ETR	1.1	1.11	1.29	1.14	1.11	1.17	1.22	1.3	1.29	1.11
Mean VIF	1.14	1.4	1.56	1.35	1.2	1.16	1.46	1.58	1.47	1.21

Source: Authors’ computations. Notes: For the definition of variables, please see Table 4.

**Table 9:** The outcomes of panel data pooled regression models regarding the influence of water, waste, and energy consumption, alongside firm and corporate governance-specific variables, on price-to-book value.

Variables	Model 1	Model 2	Model 3	Model 4	Model 5
WOMFRC	0.18	0.17	0.16	0.09	0.02
	(0.0589) **	(0.0708) **	(0.0835)	(0.0403) **	(0.0353)
	(0.0471) **	(0.0602) **	(0.0794) **	(0.0399) **	(0.0311)
EXECOMP	−0.03	−0.01	0.00		
	(0.0101) **	(0.0126) **	(0.0255)		
	(0.0072) **	(0.0088)	(0.0132)		
TDC	0.08	0.11		0.09	
	(0.0253) **	(0.0314) **		(0.0213) **	
	(0.0278) **	(0.0339) **		(0.0312) **	
CURR	−0.56	−0.45	0.38	−0.08	
	(0.3147)	(0.3776)	(0.3839)	(0.2717)	
	(0.3795)	(0.4655)	(0.4229)	(0.1584)	
DY	−1.13	−1.37	−1.08	−0.84	
	(0.1923) **	(0.2087) **	(0.2131) **	(0.1182) **	
	(0.1648) **	(0.2234) **	(0.1840) **	(0.1676) **	
OM	0.20			0.11	
	(0.0438) **			(0.0506) **	
	(0.0534) **			(0.0345) **	
ETR	0.07	0.07	0.07	0.07	0.00
	(0.0039) **	(0.0044) **	(0.0045) **	(0.0024) **	(0.0260)
	(0.0017) **	(0.0020) **	(0.0017) **	(0.0021) **	(0.0215)
CFFI		−0.00			
		(0.0000)			
		(0.0000)			
TA		−0.00	−0.00	−0.00	0.00
		(0.0000)	(0.0000)	(0.0000)	(0.0000) **
		(0.0000)	(0.0000)	(0.0000)	(0.0000) **
EMP		−0.00			
		(0.0000)			
		(0.0000)			
TWU			−0.00		−0.00
			(0.0000)		(0.0000)
			(0.0000) **		(0.0000)
CFFF			−0.00		
			(0.0000)		
			(0.0000) **		
TEC				0.00	
				(0.0001)	
				(0.0001)	
TW				−0.00	−0.00
				(0.0003)	(0.0003)
				(0.0001) **	(0.0000) **
BRDCOMP					0.00
					(0.1412)
					(0.1553)
TDTA					0.01
					(0.0240)
					(0.0307)
QR					0.03
					(0.2245)
					(0.2126)
DPR					−0.00
					(0.0103)
					(0.0078)
_cons	−2.15	2.75	1.31	−3.00	2.39
	(2.5864)	(3.1134)	(3.4795)	(1.9369)	(1.7959)
	(1.8461)	(2.1157)	(2.5209)	(1.5941)	(1.7943)
R−sq	0.8272	0.8011	0.8461	0.9465	0.1117
Obs.	112	108	78	82	102

Source: Authors’ computations. Notes: Superscripts ** indicate a statistical significance level of 5%. The first figure between brackets shows the standard deviation, while the second figure between brackets shows the robust standard deviation. For the definition of variables, please see Table 4.

**Table 10:** VIFs for the panel data pooled regression models regarding the influence of water, waste, and energy consumption, alongside firm and corporate governance-specific variables, on price-to-book value.

Variables	Model 1	Model 2	Model 3	Model 4	Model 5
WOMFRC	1.14	1.21	1.18	1.26	1.54
TWU			1.03		1.08
TEC				1.6	
TW				1.04	1.03
EXECOMP	1.09	1.38	2.02		
BRDCOMP					1.17
CFFF			1.91		
CFFI		1.72			
TDC	1.16	1.32		1.49	
TDTA					1.16
CURR	1.2	1.4	1.27	1.58	
QR					1.42
DY	1.13	1.06	1.06	1.11	
DPR					1.16
EMP		1.32			
TA		2.49	2.84	1.72	1.22
OM	1.2			2.11	
ETR	1.17	1.22	1.3	1.29	1.11
Mean VIF	1.15	1.46	1.58	1.47	1.21

Source: Authors’ computations. Notes: For the definition of variables, please see Table 4.

**Table 11:** The outcomes of panel data pooled regression models regarding the influence of water, waste, and energy consumption, alongside firm and corporate governance-specific variables, on return on capital and return on invested capital.

Variables	ROC					ROIC				
	Model 1	Model 2	Model 3	Model 4	Model 5	Model 1	Model 2	Model 3	Model 4	Model 5
WOMFRC	0.37	0.37	0.36	0.12	0.03	0.47	0.41	0.48	0.27	0.06
	(0.1672) **	(0.1701) **	(0.1987)	(0.1571)	(0.1401)	(0.1639) **	(0.1604) **	(0.1845) **	(0.1311) **	(0.1307)
	(0.1284) **	(0.1227) **	(0.1573) **	(0.1171)	(0.1320)	(0.1306) **	(0.1212) **	(0.1833) **	(0.1137) **	(0.1244)
EXECOMP	−0.04	−0.01	0.06			−0.05	−0.01	0.05		
	(0.0291)	(0.0315)	(0.0615)			(0.0285)	(0.0297)	(0.0570)		
	(0.0126) **	(0.0178)	(0.0345)			(0.0132) **	(0.0154)	(0.0332)		
TDC	−0.08	−0.04		0.03		−0.13	−0.08		−0.01	
	(0.0393) **	(0.0389)		(0.0376)		(0.0386) **	(0.0367) **		(0.0312)	
	(0.0721)	(0.0678)		(0.0837)		(0.0584) **	(0.0473)		(0.0591)	
CURR	−2.91	−1.46	−0.29	−2.05		−2.78	−1.36	0.04	−2.16	
	(0.9154) **	(0.9339)	(0.8992)	(1.0740)		(0.8976) **	(0.8823)	(0.8323)	(0.8961) **	
	(0.8661) **	(0.7683)	(0.8071)	(1.0348)		(0.8769) **	(0.7567)	(0.7087)	(1.0156) **	
DY	−0.12	−0.17	0.35	0.52		−1.70	−1.74	−1.31	−1.15	
	(0.5579)	(0.5179)	(0.5143)	(0.4771)		(0.5468) **	(0.4887) **	(0.4776) **	(0.3963) **	
	(0.8514)	(0.9433)	(0.7308)	(0.6667)		(0.4272) **	(0.5050) **	(0.3500) **	(0.2775) **	
OM	0.24			0.52		0.18			0.50	
	(0.1286)			(0.2018) **		(0.1237)			(0.1591) **	
	(0.1373)			(0.1468) **		(0.1331)			(0.1038) **	
ETR	−0.03	−0.04	−0.04	−0.04	−0.25	−0.02	−0.02	−0.03	−0.02	−0.23
	(0.0110) **	(0.0104) **	(0.0108) **	(0.0092) **	(0.1033) **	(0.0108)	(0.0099) **	(0.0100) **	(0.0077) **	(0.0968) **
	(0.0052) **	(0.0052) **	(0.0035) **	(0.0047) **	(0.0742) **	(0.0035) **	(0.0032) **	(0.0029) **	(0.0031) **	(0.0780) **
CFFI		−0.00					−0.00			
		(0.0001)					(0.0001)			
		(0.0001)					(0.0001)			
TA		−0.00	−0.00	−0.00	0.00		−0.00	−0.00	−0.00	0.00
		(0.0000)	(0.0000)	(0.0000)	(0.0000)		(0.0000) **	(0.0000)	(0.0000)	(0.0000)
		(0.0000)	(0.0000)	(0.0000)	(0.0000) **		(0.0000) **	(0.0000)	(0.0000)	(0.0000) **
EMP		0.00					0.00			
		(0.0000) **					(0.0000) **			
		(0.0000) **					(0.0000) **			
TWU			−0.00		0.00			−0.00		0.00
			(0.0000)		(0.0000)			(0.0000)		(0.0000)
			(0.0000)		(0.0000)			(0.0000)		(0.0000)
CFFF			−0.00					−0.00		
			(0.0001)					(0.0001)		
			(0.0000)					(0.0000)		
TEC				−0.00					−0.00	
				(0.0006)					(0.0005)	
				(0.0005)					(0.0005)	
TW				−0.00	−0.00				−0.00	−0.00
				(0.0015)	(0.0013)				(0.0012)	(0.0012)
				(0.0004) **	(0.0004) **				(0.0004) **	(0.0004) **
BRDCOMP					0.61					0.01
					(0.6875)					(0.5303)
					(0.8142)					(0.5104)
TDTA					−0.11					−0.20
					(0.0890)					(0.0828) **
					(0.1016)					(0.1186)
QR					−1.48					−1.13
					(0.8823)					(0.8261)
					(1.3258)					(1.1071)
DPR					−0.02					−0.01
					(0.0416)					(0.0388)
					(0.0306)					(0.0375)
_cons	21.23	18.46	8.74	8.17	24.32	23.43	20.96	6.98	11.17	24.16
	(7.3841) **	(7.5033) **	(7.9734)	(7.8089)	(7.3724) **	(7.1781) **	(7.0694) **	(7.4198)	(6.4248) **	(6.6946) **
	(6.1626) **	(4.9834) **	(5.9823)	(5.9090)	(8.4967) **	(6.1727) **	(5.0084) **	(5.9719)	(5.2921) **	(7.4999) **
R−sq	0.3102	0.3308	0.2894	0.3278	0.1874	0.3659	0.4055	0.3131	0.3865	0.1927
Obs.	116	111	81	85	104	120	115	84	88	107

Source: Authors’ computations. Notes: Superscripts ** indicate a statistical significance level of 5%. The first figure between brackets shows the standard deviation, while the second figure between brackets shows the robust standard deviation. For the definition of variables, please see Table 4.

**Table 12:** VIFs for the panel data pooled regression models regarding the influence of water, waste, and energy consumption, alongside firm and corporate governance-specific variables, on return on capital and return on invested capital.

Variables	ROC					ROIC				
	Model 1	Model 2	Model 3	Model 4	Model 5	Model 1	Model 2	Model 3	Model 4	Model 5
WOMFRC	1.14	1.19	1.16	1.18	1.54	1.14	1.2	1.17	1.19	1.51
TWU			1.03		1.09			1.03		1.08
TEC				1.57					1.55	
TW				1.04	1.03				1.04	1.03
EXECOMP	1.07	1.39	2.02			1.07	1.39	2.04		
BRDCOMP				1.2					1.17	
CFFF			1.95					1.9		
CFFI		1.65					1.65			
TDC	1.09	1.15		1.19		1.09	1.15		1.18	
TDTA					1.21					1.19
CURR	1.21	1.4	1.21	1.51		1.23	1.43	1.22	1.54	
QR					1.37					1.39
DY	1.12	1.06	1.06	1.1		1.12	1.06	1.06	1.09	
DPR					1.15					1.16
EMP		1.3					1.34			
TA		2.26	2.81	1.6	1.18		2.27	2.81	1.59	1.18
OM	1.22			2.02		1.22			1.89	
ETR	1.1	1.11	1.29	1.14	1.11	1.1	1.11	1.29	1.14	1.11
Mean VIF	1.14	1.39	1.57	1.37	1.21	1.14	1.4	1.56	1.35	1.2

Source: Authors’ computations. Notes: For the definition of variables, please see Table 4.
